# *In Vivo* Transcriptome of Lactobacillus acidophilus and Colonization Impact on Murine Host Intestinal Gene Expression

**DOI:** 10.1128/mBio.03399-20

**Published:** 2021-01-26

**Authors:** Yong Jun Goh, Rodolphe Barrangou, Todd R. Klaenhammer

**Affiliations:** a Department of Food, Bioprocessing and Nutrition Sciences, North Carolina State University, Raleigh, North Carolina, USA; University of Michigan Medical School

**Keywords:** *Lactobacillus*, acidophilus, gut adaptation, *in vivo* gene expression, mouse colonization, probiotic

## Abstract

Lactobacillus acidophilus NCFM is a probiotic strain commonly used in dairy products and dietary supplements. Postgenome *in vitro* studies of NCFM thus far have linked potential key genotypes to its probiotic-relevant attributes, including gut survival, prebiotic utilization, host interactions, and immunomodulatory activities. To corroborate and extend beyond previous *in vivo* and *in vitro* functional studies, we employed a dual RNA sequencing (RNA-seq) transcriptomic approach to identify genes potentially driving the gut fitness and activities of L. acidophilus NCFM *in vivo*, and in parallel, examine the ileal transcriptional response of its murine hosts during monocolonization. Spatial expression profiling of NCFM from the ileum through the colon revealed a set of 134 core genes that were consistently overexpressed during gut transit. These *in vivo* core genes are predominantly involved in the metabolism of carbohydrates, amino acids, and nucleotides, along with mucus-binding proteins and adhesion factors, confirming their functionally important roles in nutrient acquisition and gut retention. Functional characterization of the highly expressed major S-layer-encoding gene established its indispensable role as a cell shape determinant and maintenance of cell surface integrity, essential for viability and probiotic attributes. Host colonization by L. acidophilus resulted in significant downregulation of several proinflammatory cytokines and tight junction proteins. Genes related to redox signaling, mucin glycosylation, and circadian rhythm modulation were induced, suggesting impacts on intestinal development and immune functions. Metagenomic analysis of NCFM populations postcolonization demonstrated the genomic stability of L. acidophilus as a gut transient and further established its safety as a probiotic and biotherapeutic delivery platform.

## Abstract (precis)

To date, our basis for comprehending the probiotic mechanisms of Lactobacillus acidophilus, one of the most widely consumed probiotic microbes, was largely limited to *in vitro* functional genomic studies. Using a germfree murine colonization model, *in vivo*-based transcriptional studies provided the first view of how L. acidophilus survives in the mammalian gut environment, including gene expression patterns linked to survival, efficient nutrient acquisition, stress adaptation, and host interactions.

## INTRODUCTION

Lactobacillus acidophilus is one of the most commercially significant microbial species, with several strains commonly used as probiotic cultures worldwide in the dairy and dietary supplement industries, owing to its long safety history and proposed health-promoting effects, along with its amenability to industrial production (1, 2). An indigenous member of the human gastrointestinal microbiota (3, 4), survival of L. acidophilus in the gut environment relies on its adaptive mechanisms to extreme conditions, including pH fluctuations, the presence of bile acids, antimicrobial compounds, and hydrolytic enzymes, and its ability to utilize a broad repertoire of carbohydrates and other nutrient sources in the gut. L. acidophilus NCFM was originally isolated from a human and commercialized since the early 1970s (1, 4). Concomitantly, NCFM has emerged as one of the most characterized commercial probiotic strains documented for its health-promoting attributes (1), such as the alleviation of cold and influenza-like symptoms (5), modulation of immune cell functions (6), and moderation of visceral pain receptors (7). Due to its generally regarded as safe (GRAS) status and the ability to survive transit through the gastrointestinal tract (GIT), along with its genetic amenability (8, 9), NCFM is increasingly becoming an attractive vehicle for mucosa-targeted delivery of vaccines and biotherapeutics (10, 11).

The release of the NCFM genome in 2005 (12) has served as a blueprint for uncovering the key genotypes and molecular mechanisms involved in the adaptation and host interactions of this species in the GIT. Functional studies *in vitro* have revealed many genetic determinants contributing to its survival and health-promoting attributes in the gut. These determinants include acid and bile tolerance (13, 14), epithelial adhesion and immunomodulation (15–17), prebiotic carbohydrate utilization (18–20), bioconversion of polyphenols (21), and *in vivo* competitive colonization studies substantiating the importance of glycogen metabolism and sortase-dependent cell surface proteins in gut persistence (22, 23). Nevertheless, *in vivo* studies were lacking to support the predicted genomic features and adaptive responses that define the lifestyle and fitness of this probiotic microbe in the gut.

A number of studies have reported the *in vivo* adaptive response and colonization determinants of lactobacilli in the gut. In Lactobacillus reuteri, a combination of *in vivo* expression technology (IVET) and competitive colonization studies have established the functional roles of the Lsp surface protein and a methionine sulfoxide reductase on the gut performance of L. reuteri in reconstituted *Lactobacillus*-free mice (24, 25). Microarray expression analysis of Lactobacillus plantarum colonized in the ceca of gnotobiotic mice revealed carbohydrate transport and metabolism as the main upregulated functional group. Remarkably, more than half of the induced genes are located in the “lifestyle adaptation region” of L. plantarum encoding glycan transport and degradation, reported to reflect host adaptation strategies through efficient metabolism of diet- and host-derived carbohydrates (26). Comparative transcriptomes with *L. plantarum* strain 299v (isolated from human biopsy specimens from the ileum and colon) further showed expression profiles similar to those of strain WCFS1 in mono-associated mice fed a western diet. The convergence of these transcriptome data validate the relevance of germfree murine models in examining the adaptive response of lactobacilli in gut environments (27). In Lactobacillus johnsonii, a combinatory genotyping and expression profiling approach comparing strain NCC533 with the neotype strain identified a mannose phosphotransferase system (PTS) and a putative immunoglobulin A protease which contributed to its gut persistence phenotype (28).

Here, we aimed to investigate the intestinal exposure of L. acidophilus NCFM via transcriptome studies during gut transit in gnotobiotic mice. Concurrently, we also examined the gene expression of intestinal tissues of NCFM-colonized mice to explore the molecular responses induced by administration of NCFM. Considering genome stability as an important attribute of a probiotic and as a biotherapeutic delivery vehicle, we further assessed the genome integrity of NCFM by metagenome sequencing postcolonization and following transit through the GIT.

## RESULTS AND DISCUSSION

**FIG 1:**
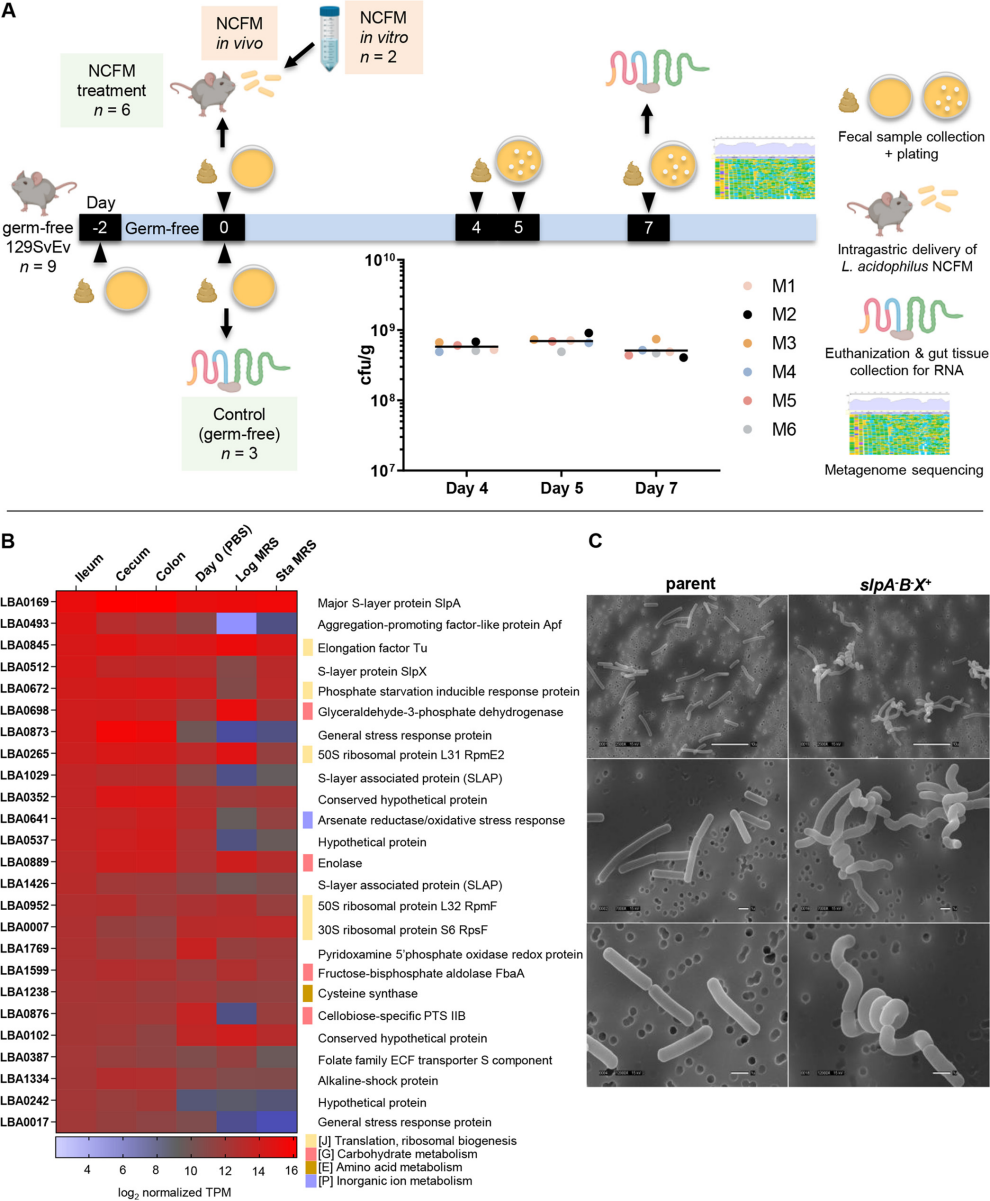
L. acidophilus NCFM colonization studies and identification of highly expressed genes *in vivo*. (A) For murine colonization experimental design, 2 days prior to the study (day −2), nine germfree 129S6/SvEv mice (24 to 25 weeks old) were verified germfree by culturing fecal samples aerobically and anaerobically on plate count agar and MRS agar prior to the experiments. On the start day of experiment (day 0), three germfree mice (control group without NCFM treatment) were removed from the isolator and euthanized, and intestinal tissues from the duodenum, jejunum, ileum, cecum, and colon were harvested for RNA isolation. In parallel, stationary-phase NCFM cells grown in MRS broth were washed once with phosphate-buffered saline (PBS) (pH 7.4), resuspended in PBS, and administered to the remaining six mice (ca. 6 × 10^8^ CFU in 200 μl per mouse) in the experimental group by intragastric gavage. For NCFM *in vitro* control, cell aliquots from the same PBS suspension were collected for RNA isolation. Fecal samples from all six NCFM-fed mice were collected on days 4, 5, and 7 for plating and enumeration of NCFM to assess gut colonization status (see the inset scatterplot of fecal bacterial count from all six mice, mouse 1 [M1] to M6). On day 7, all six mice were euthanized, and tissue compartments were harvested as described above for RNA isolation. Portions of the six fecal samples collected on day 7 were also cultured in MRS broth for metagenome sequencing. (B) Highest transcribed genes (top 25, in decreasing order) in L. acidophilus NCFM isolated from the ileum and respective expression levels in the cecum and colon, as well as in PBS suspension prior to gavage, and log- and stationary-growth phases in MRS medium (Log MRS and Sta MRS, respectively). Genes belonging to specific COG functional categories are indicated. ECF, energy-coupling factor. (C) SEM imaging of NCFM demonstrating the major impacts of inactivating the highest expressed gene, *slpA*, on the morphological changes in the *slpAB slpX^+^* (NCK1973B) double mutant. The mutant cells lost the species-defining rod shape and appeared as tight spirals or curly rod conformations. SEM images were captured at 2,500×, 7,000×, and 12,000× magnifications.

Considering the distinctive physiological conditions along the longitudinal axis of the GIT (e.g., pH, oxygen, osmolarity gradient, bile acids, and antimicrobial peptides), transcriptional profiling of L. acidophilus NCFM residing in the small and large intestines allowed us to examine responses that may contribute to ecological fitness of L. acidophilus during transit through the GIT. L. acidophilus NCFM was administered to germfree 129S6/SvEv mice, and colonization was confirmed on day 4 (23) at 5.79 ×10^8^ ± 8.23 × 10^7^ CFU/g (Fig. 1A). On day 7, mice were euthanized, and the intestinal sections (duodenum, jejunum, ileum, cecum, and colon) were harvested for RNA isolation. Bacterial total RNA was obtained from the ileum, cecum, and colon for mRNA sequencing analysis (see Tables S1 and S2 at Dryad, https://doi.org/10.5061/dryad.mcvdncjzr). The yield of bacterial RNA isolated from the duodenum and jejunum was too low to permit further analysis. The inherent difficulty of obtaining quality bacterial RNA from the upper intestinal tract has also been reported in previous *in vivo* transcriptome studies of L. johnsonii and *L. plantarum* (29, 30), likely due to lower bacterial density and the physiological conditions in this region.

Overall, the *in vivo* transcriptome profiles of L. acidophilus NCFM were distinctive from the *in vitro* conditions grown in MRS medium (log and stationary phases) or stationary-phase NCFM resuspended in phosphate-buffered saline (PBS) prior to gavage (see Fig. S1 at Dryad, https://doi.org/10.5061/dryad.mcvdncjzr). Under *in vivo* conditions, the transcriptome profiles were clearly niche dependent, with higher similarity in the overall transcriptome profiles between both cecal and colonic NCFM populations compared to the NCFM population from the ileum (Fig. S1 at the above URL).

### L. acidophilus NCFM genes highly expressed *in vivo*.

First, we examined the highest mRNA abundance transcribed *in vivo* to detect genes or functions that were potentially important for gut survival and adaptation of NCFM. Figure 1B highlights the top 25 highest transcribed genes in L. acidophilus NCFM from the ileum and their relative expression levels in the other sampling conditions. The major S-layer gene, *slpA*, appeared to be the highest expressed gene along the gut (see Table S3 at Dryad, https://doi.org/10.5061/dryad.mcvdncjzr). Notably, genes previously associated with adhesion to intestinal epithelial cells and stress tolerance, e.g., the aggregation-promoting factor-like protein (31), the S-layer-associated proteins (32, 33), together with several uncharacterized proteins, were highly expressed *in vivo*. Interestingly, a group of stress response proteins predicted to cope with general stress, alkaline shock/cell envelope stress, oxidative stress, and phosphate starvation were also highly upregulated *in vivo*. This reflects a stress adaptation pattern distinct from that of the canonical stress response protein families, including the Clp proteases and chaperones GroES-GroEL and DnaJ-DnaK-GrpE which dominated the most highly transcribed genes in both stationary-phase cells grown in MRS medium and cells resuspended in PBS prior to gavage into mice (day 0) (see Table S4 at Dryad, https://doi.org/10.5061/dryad.mcvdncjzr). Moreover, numerous ribosomal proteins and glycolytic enzymes were highly induced *in vivo*, suggesting that NCFM cells were metabolically active in the gut.

### S-layer: biological functions of the most highly expressed genes.

S-layers are monomolecular crystalline layers of protein or glycoprotein subunits that form the outermost envelope of archaea and some eubacteria, including some species of lactobacilli. L. acidophilus NCFM has three S-layer-encoding genes, *slpA* (*lba0169*; dominant), *slpB* (*lba0175*; silent), and *slpX* (*lba0512*; auxiliary). Analogous to laboratory growth conditions, *slpA* is the highest expressed gene *in vivo*, with *slpX* also among the most highly transcribed genes during gut transit (see Tables S3 and S4 at the above URL), prompting us to further probe the fundamental roles of this metabolically expensive cell surface layer in strain NCFM. Inactivation of *slpA* in a previous study resulted in chromosomal inversion where *slpB* was dominantly expressed in place of *slpA* in the NCFM mutant (6). During the current study, construction of an *slp* null mutant (*slpABX* mutant) to investigate the biological significance of S-layer was unsuccessful. We subsequently generated an *slpAB slpX^+^* double mutant (NCK1973B) with consecutive inactivation of *slpB* and *slpA*. Scanning electron microscopy (SEM) imaging of strain NCK1973B revealed that mutant cells lost the straight, uniform rod-shaped morphotype and appeared as coiled or spiral rods (Fig. 1C). In addition, the growth-impaired NCK1973B also exhibited increased sensitivity to bile challenge, simulated gastric juice and high osmolarity (Fig. S2A and B at the above URL). These observations translate into low survivability of NCK1973B *in vivo* and the potential protective roles of S-layer during gut transit.

SlpA and SlpB deficiencies in L. acidophilus NCK1973B did not significantly reduce adhesion to Caco-2 epithelial cells and porcine mucin, similar to previous observations with removal of SlpX (9) (see Fig. S2C at Dryad, https://doi.org/10.5061/dryad.mcvdncjzr), although the role of S-layer in epithelial adhesion *in vivo* remains to be established. *In vitro* dendritic cell (DC) cytokine profiling revealed significant induction of interleukin 6 (IL-6), IL-12, tumor necrosis factor alpha (TNF-α), as well as IL-10 by NCK1973B (see Fig. S2D at the URL above). We hypothesized that an intact S-layer acts as a shield which masks the majority of the cell surface components from overstimulating the host innate immune response. The morphologically impaired NCK1973B clearly demonstrated the fundamental roles of an intact S-layer in maintaining cell shape and integrity, and the consequential pleiotropic effects that will impact cell viability, host signaling, and probiotic functionality.

### *In vivo* core and spatial transcriptomes.

**FIG 2:**
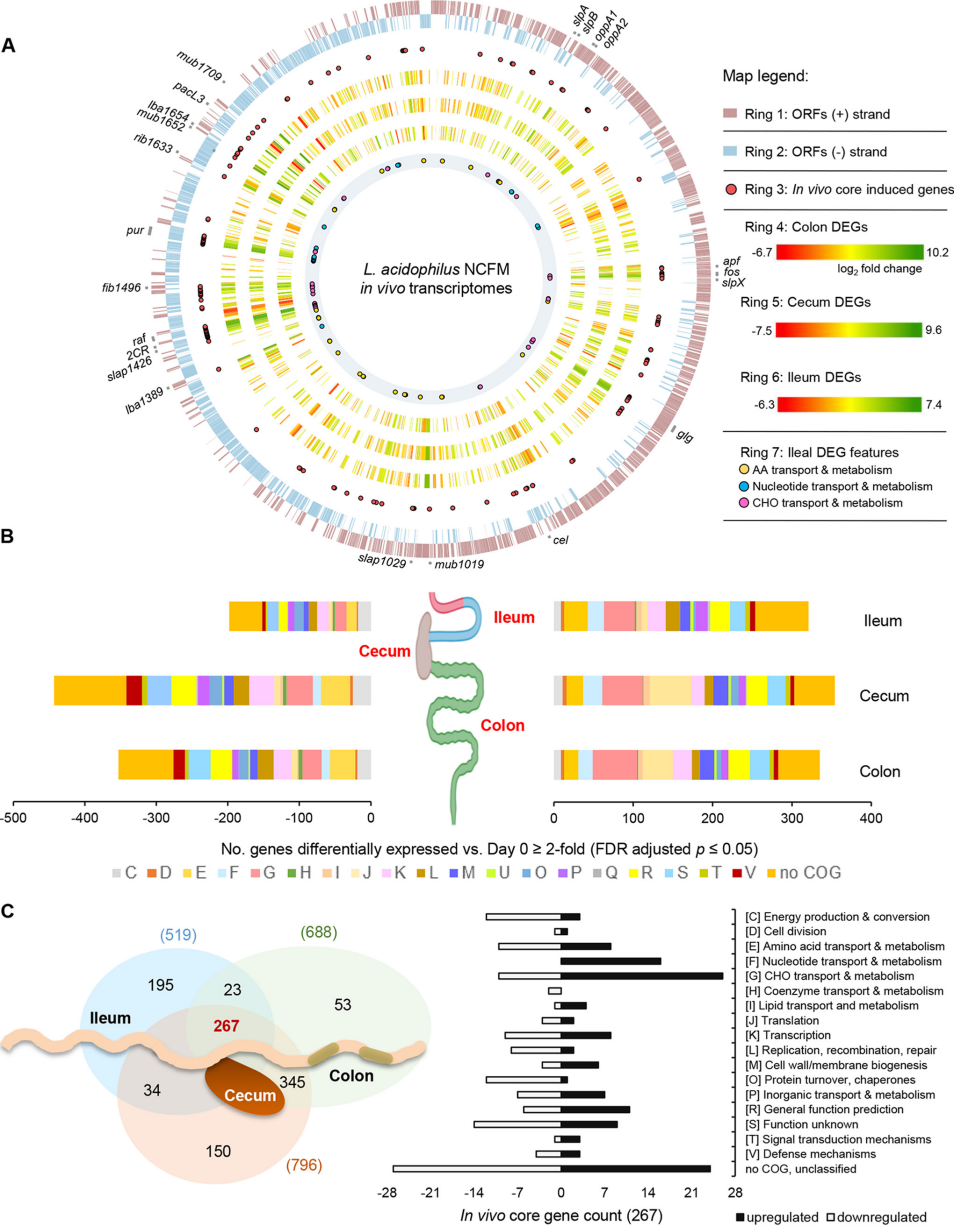
*In vivo* core and spatial transcriptomes of L. acidophilus NCFM. (A) Circular genome representation of differentially expressed genes (DEGs) in the ileum, cecum, and colon with the corresponding fold change in expression values mapped to the NCFM chromosome (GenBank accession number NC_006814) (rings 4 to 6). A core upregulated gene set (134 genes) mapped to the chromosome (ring 3, red circles) revealed the presence of gene clusters or genomic “hot spots” that were highly transcribed under *in vivo* conditions. Upregulated genes involved in carbohydrate, amino acid, and nucleotide transport and metabolism in the ileal population were represented in ring 7. Selected key gene features were highlighted in the outer ring. Genome mapping and visualization of DEGs were performed using the CiVi tool (65). ORFs, open reading frames; AA, amino acid; CHO, carbohydrate. (B) Stacked bar graph depicting the number and functional distribution of the differentially regulated genes in the ileum, cecum, and colon based on COG classification (see panel C). (C) Venn diagram illustrating spatial distribution of the differentially expressed genes in the gut niches (left), with further emphasis on the functional distribution of the core genes depicted in the bar graph (right). *fos*, FOS utilization; *glg*, glycogen metabolism; *cel*, cellobiose utilization; *2CR*, bile-inducible two-component regulatory system LBA1430-1431; *raf*, raffinose utilization; *pur*, purine biosynthesis.

The baseline transcriptional profile of L. acidophilus NCFM was measured from stationary-phase cells resuspended in PBS prior to oral gavage (*in vitro* control, representing initial/zero time point baseline for measuring transcriptional changes following *in vivo* colonization; Fig. 1A). The highest number of differentially expressed genes *in vivo* (≥2-fold change in normalized transcripts per million [TPM], false discovery rate [FDR]-adjusted *P* value < 0.05) were observed in NCFM residing in the cecum (796 [41%] of the total number of open reading frames in the genome, or ORFeome), followed by colon (688 [36%]) and ileum (519 [27%]) (Fig. 2). Overall, the majority of the highly upregulated genes *in vivo* were heavily skewed toward functional categories involving carbohydrate (G), amino acid (E) and nucleotide (F) metabolism, transcription (K), replication and repair functions (L), along with numerous unknown proteins (R, S) and proteins with no clusters of orthologous groups of proteins (COG) designation (Fig. 2B), the latter of which encompasses the majority of cell surface proteins.

### *In vivo* core genes.

A set of 267 genes (14% of ORFeome), designated the “*in vivo* core genes” were differentially expressed in L. acidophilus NCFM during transit along the gut (see Table S5 at Dryad, https://doi.org/10.5061/dryad.mcvdncjzr), representing 51%, 34%, and 39% of the differentially regulated genes in the microbe when residing in the ileum, cecum, and colon, respectively (Fig. 2C). Interestingly, some of the core induced genes appeared to concentrate at specific chromosomal regions (e.g., on minus strand near the origin of replication) or were encoded in operons (Fig. 2A, ring 3). The coregulation of genes located within these genomic hot spots or potential gut-adaptive genomic islands likely enabled dynamic response of NCFM to nutrient availability and environmental changes that may provide a competitive advantage and reflect the specialized adaptation of NCFM to the mammalian gut similar to that observed in *L. plantarum* (26).

**FIG 3:**
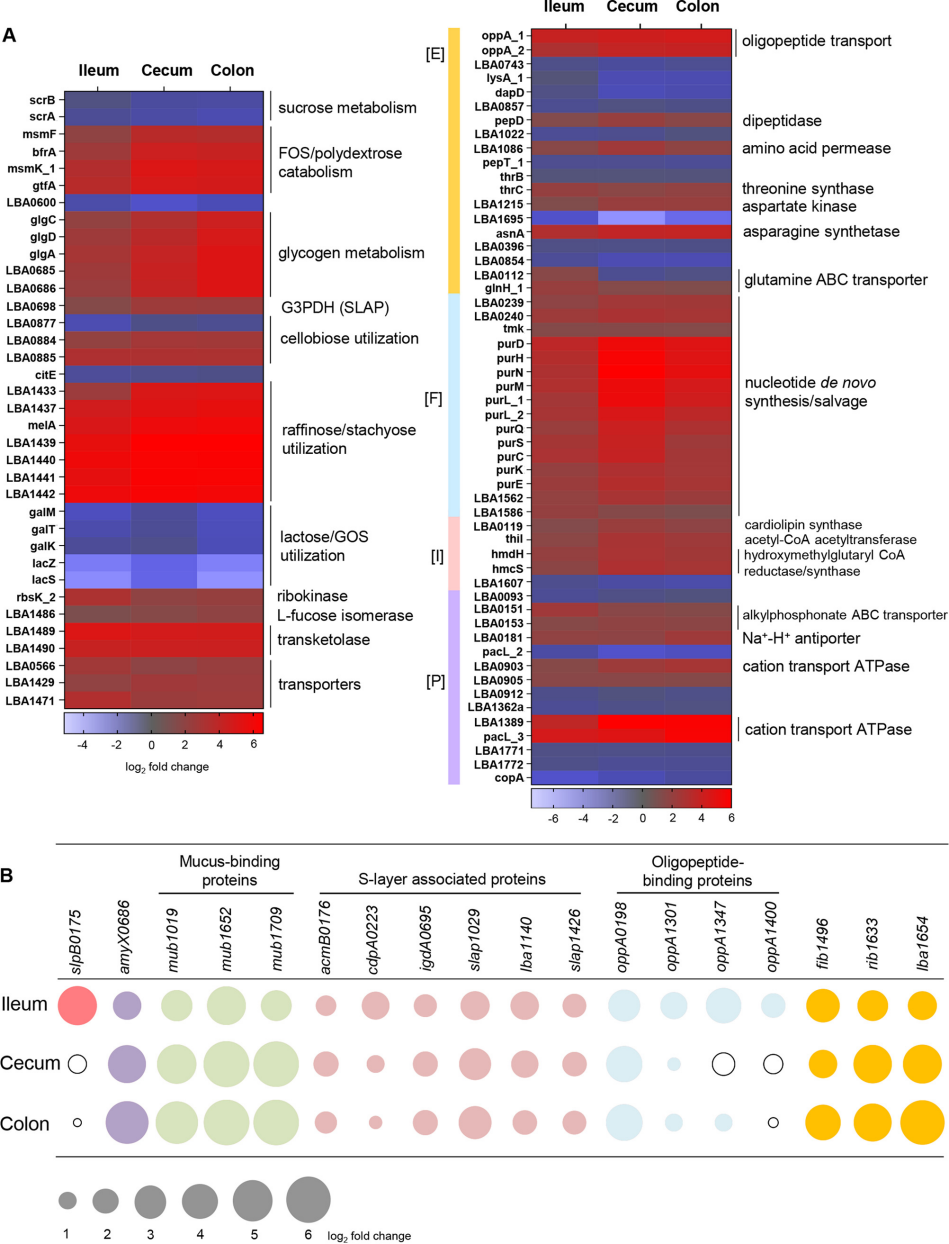
(A) *In vivo*, differentially regulated core genes in L. acidophilus NCFM involved in carbohydrate [G] (left panel), amino acid [E], nucleotide [F], lipid [I], and inorganic ion transport and metabolism [P] (right panel) (see Table S5 at Dryad, https://doi.org/10.5061/dryad.mcvdncjzr). (B) Ileum-induced genes of cell surface proteins and their respective expression levels in the cecum and colon. Fold change of induction corresponds to bubble size, and downregulated fold change is represented by unfilled bubbles. Log_2_ fold change, differential expression ratio in ileum, cecum, or colon versus stationary-phase cells resuspended in PBS prior to oral gavage (baseline transcriptome on day 0, *in vitro*). G3PDH, glyceraldehyde-3-phosphate dehydrogenase; CoA, coenzyme A.

The core induced genes were composed predominantly of those involved in the transport and metabolism of carbohydrates, proteins, and nucleotides (Fig. 2C and 3A), indicating their pivotal roles in host survival and nutrient acquisition. Moreover, additional core genes induced were putative surface adhesins, mucus-binding proteins, and S-layer-associated proteins that are potentially involved in host adhesion and immunomodulatory functions (Fig. 3B). Among these are five sortase-dependent LPxTG cell wall-anchored proteins, including two mucus-binding proteins (LBA1019 and LBA1652), a putative fibrinogen-binding protein (LBA1496), and two unknown surface proteins (LBA1654 and LBA1633) (Fig. 3B and 2A). Genes encoding LBA1652, LBA1496, and LBA1654 were previously found to be overexpressed upon bile exposure *in vitro* (14). LBA1633 contains multiple copies of Rib/alpha domains (PF08428), also found in surface proteins in other lactobacilli which have been associated with adhesion to cervical and vaginal epithelia (34, 35). Our previous study demonstrated the negative impact of sortase inactivation on gut retention of strain NCFM *in vivo* (23). The induction of these five LPxTG-anchored proteins pinpoints the potential roles of these specific sortase-dependent proteins on host interactions of NCFM in the GIT.

The gene clusters previously associated with the utilization of raffinose, stachyose, fructooligosaccharide (FOS), polydextrose, and cellobiose (18, 20) and glycogen metabolism (36) were consistently upregulated along the gut (Fig. 3A). Induction of genes for FOS, raffinose, and stachyose catabolism could be attributed to the presence of these nondigestible oligosaccharides in the mouse chow (37). We first reported the biological importance of glycogen metabolic pathways in L. acidophilus NCFM, where similar pathways were also found primarily in *Lactobacillus* species commonly associated with natural or mammalian host environments (36). Inactivation of the pathways negatively impacted growth, carbon utilization capability, bile tolerance, and more importantly, the *in vivo* competitiveness of NCFM in murine gut (22, 36). The consistent upregulation of this metabolic pathway during gut transit further established the significance of glycogen storage and carbon cycling on the gut fitness of NCFM. As the principal functional group of genes most impacted by *in vivo* colonization, the induction of carbohydrate metabolic genes during *in vivo* transit appears to be a signature expression pattern among gut-colonized lactobacilli, including *L. plantarum* (26) and L. johnsonii (28, 30).

The overrepresentation of *de novo* nucleotide biosynthesis and salvage pathways genes (12% of induced core genes; Fig. 3A) highlights the importance of nucleotide biosynthesis in colonization of the murine gut. In other bacteria, the ability to synthesize nucleotides is a major prerequisite for colonization and pathogenesis (38, 39). One of the major facilitator superfamily (MFS) transporters (LBA1429; Fig. 3A) and its associated bile-inducible operon previously implicated in bile tolerance (14) were consistently coupregulated *in vivo.* Interestingly, three cation-transporting P-type ATPases were also upregulated (Fig. 3A). Members of this P-type ATPase family are involved in translocating cations to maintain membrane electrochemical gradients, e.g., during alkaline pH stress, scavenging of trace elements, and export of toxic heavy metals.

### Ileum-specific genes.

Comparison of the niche-specific transcriptomes of L. acidophilus NCFM revealed a large subset of differentially regulated genes unique to the ileal population (195/519 or 38%; Fig. 2C) (see Table S6 at Dryad, https://doi.org/10.5061/dryad.mcvdncjzr) compared to those in the cecum and colon (150/796 or 19%, and 53/688 or 8%, respectively). Approximately 73% (142/195) of ileum-specific genes are involved in amino acid transport and metabolism, transcriptional regulation, replication and recombination, and proteins of unknown functions. In addition to the five amino acid and peptide transporters in the core induced gene set, another 11 protein transport systems were also specifically upregulated in the ileum. In contrast, only 2 of the 10 predicted amino acids *de novo* pathways (pyruvate to serine, LBA1397-1398; aspartate to asparagine, LBA1896) were induced in the ileum. Altogether, these findings highlight the importance of amino acid and peptide acquisition in the small intestine. The upregulation of these transport systems would enable scavenging of these substrates that have escaped host absorption or that are physiologically abundant in the intestinal environment, e.g., glutamine (40). In addition to nutrient scavenging, it is noteworthy that the OppA substrate-binding component of oligopeptide transport systems has also been implicated in bile tolerance by binding to bile salts and preventing their cell entry (41).

Starch is the main digestible carbohydrate component in the mouse diet, with processed wheat and corn being primary chow ingredients. There was a preferential upregulation of catabolic machinery that targeted α-1,4-/α-1,6-glucoside (LBA1866-1867, LBA0264) and β-glucoside (LBA1575-1576, LBA1706-1707) substrates in the ileum, reflecting the ability of L. acidophilus NCFM to harvest carbon source from starch components partially degraded by host enzymes at the upper GIT. This included an ABC transporter permease of a maltose utilization operon which has been functionally correlated with metabolism of the prebiotics isomaltooligosaccharides, dextran, and maltotetraose (42). A putative α-l-rhamnosidase (LBA1473) was also specifically induced. Purified α-l-rhamnosidase of L. acidophilus was capable of hydrolyzing rhamnosides from the plant flavonoids rutin and naringin (43). This enzyme would potentially enable NCFM to catabolize rhamnosides derived from polyphenols commonly present in dietary plant materials.

The overexpression of the secondary Slp gene, *slpB*, of L. acidophilus NCFM specifically in the ileum was intriguing, considering no significant *slpB* expression was detected during planktonic growth (9). In other S-layer formers such as *Campylobacter* and *Clostridium*, S-layer switching plays an important role in antigenic variation and the evasion of host defense mechanisms (44, 45). The genomic architecture of the *slp* locus in NCFM allows for condition-dependent chromosomal inversion (switching) events to favor expression of *slpB* in place of *slpA* (46). The present transcriptome data showed S-layer switching in a subpopulation of NCFM in the small intestine, the prime site of the host immune system. The SlpA of NCFM modulates DCs and T cell functions by serving as a ligand that directly binds to the DC-specific ICAM-3-grabbing nonintegrin (DC-SIGN) receptor (6). When exposed to DCs, native SlpA-dominant NCFM elicited production of IL-10 and lowered production of proinflammatory cytokine IL-12p70. Conversely, a *slpA* knockout strain that expresses only *slpB* demonstrated significantly reduced binding to DCs and induced an altered pattern of cytokine production, specifically inducing IL-12p70, TNF-α, and IL-1β (6). Nevertheless, *slpA* remained the highest expressed gene *in vivo*, indicating that the majority of the NCFM population in the ileum are SlpA dominant. We speculate that the subpopulation of the *slpB*-expressing NCFM could be host triggered for S-layer switching from SlpA→SlpB as a mechanism to establish immune tolerance.

### Shared NCFM genes expressed in cecum and colon.

The cecal and colonic NCFM populations shared 345 differentially expressed genes that were strikingly also coordinately regulated in both niches (see Table S7 at Dryad, https://doi.org/10.5061/dryad.mcvdncjzr). Overall, the transcriptome profiles of L. acidophilus NCFM in the cecum and colon were highly overlapped, with 77 to 89% of the differentially expressed genes (including *in vivo* core genes) coregulated in both niches (Fig. 2C). These findings are unexpected but plausible considering that the cecum may serve as an upstream reservoir for the bacterial load prior to transit through the colon. The major functional categories among the shared upregulated genes (44% of 345) involved sugar PTS systems, glycolysis enzymes, cell wall biogenesis, cell division, and protein translation. This suggests that NCFM bacteria were more metabolically active and were replicating, as evidenced by the higher luminal and mucosa-associated NCFM cell densities observed previously in the cecum compared to the small intestine (23). Besides the carbohydrate catabolic machineries in the core induced gene set, notably several PTS transporters and enzymes were also induced that were associated with prebiotic α- and β-glucoside catabolism (20) and bioconversion of dietary plant glycosides with conjugated phytochemicals (LBA0227 and LBA0726) (21). The upregulation of genes related to glycerol uptake (LBA1436) is consistent with previous observations (26, 47), suggesting that NCFM is capable of utilizing glycerol derived from dietary triglycerides as an energy source and precursor for cell membrane biogenesis. Interestingly, we also observed specific upregulation of the lipoteichoic acid (LTA) biosynthesis genes (LBA0445-0447) in both cecum and colon (Table S7 at the above URL), suggesting a spatial expression pattern of this immunogenic ligand along the gut.

### Host response to NCFM colonization.

**FIG 4:**
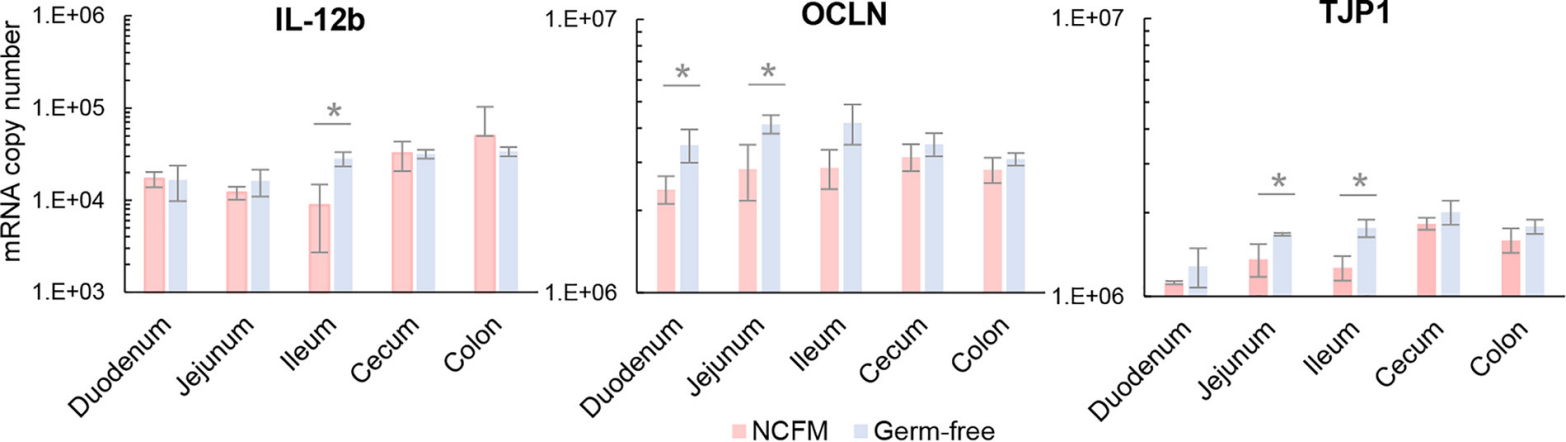
Gene expression of host-intestinal tissues in response to L. acidophilus NCFM colonization. IL-12b, OCLN, and TJP1 genes were significantly downregulated in small intestinal regions of NCFM-colonized mice (*n *=* *3) compared to germfree mice (*n *=* *3). The data represent the means of three biological replicates, and the error bars represent the standard deviations (SD). Values that are statistically significantly different (*P* < 0.05) are indicated by a bar and asterisk. No significant difference in the ACTB housekeeping gene transcript levels was observed for all tissue segments between the two groups (see Fig. S3A at Dryad, https://doi.org/10.5061/dryad.mcvdncjzr).

Parallel host transcriptional studies were conducted to examine the impact of NCFM colonization on intestinal tissue expression. Reverse transcription-quantitative PCR (RT-qPCR) analysis was initially performed on gut tissue samples from NCFM-colonized and germfree mice (*n *=* *3 per group, 2 males and 1 female; see Table S1 at Dryad, https://doi.org/10.5061/dryad.mcvdncjzr) to target expression of selected immune markers and epithelial barrier junction proteins along the gut (see Table S8 at the above URL). In NCFM-colonized mice, IL-12b, OCLN, and TJP1 genes were significantly downregulated compared to germfree mice, solely in small intestinal regions (Fig. 4). No significant change in expression was observed for the other cytokines tested, suggesting that L. acidophilus NCFM colonization did not significantly impact the native cytokine profiles of its gnotobiotic host. On the other hand, L. acidophilus L36 was previously shown to induce IL-6 and TNF-α in germfree Swiss NIH mice (48), implying a strain-dependent, and possibly host model-dependent response. The downregulation of IL-12b in the present study may be indicative of an anti-inflammatory response conferred by interaction of NCFM with the host epithelium. Hayes and colleagues (49) previously reported a decrease in colonic paracellular permeability along with higher mRNA expression of OCLN and a nonstatistically significant trend of higher ZO-1 mRNA expression in germfree mice compared to conventional C57BL/6 mice. Postcolonization of germfree mice with human fecal microbiota diminished ZO-1 expression, although with no significant change in the ZO-1 and occludin protein levels. Concomitantly, commensal colonization induced colonic paracellular permeability and lower claudin-1 protein expression resembling the physiological state in conventional mice, indicating a role of microbiota in establishing colonic barrier permeability functions (49). Considering our present study also showed a decrease in OCLN and TJP1 mRNA expression following NCFM colonization, we speculated a similar scenario occurred in the NCFM-monocolonized mice. Further work involving structural immunohistochemistry and tight junction protein expression is warranted to establish the potential role of NCFM in restoring intestinal permeability functions. Overall, the significant transcriptional changes specifically in the small intestine highlighted this region as the primary effector site where NCFM modulates its host immune and intestinal barrier functions.

**FIG 5:**
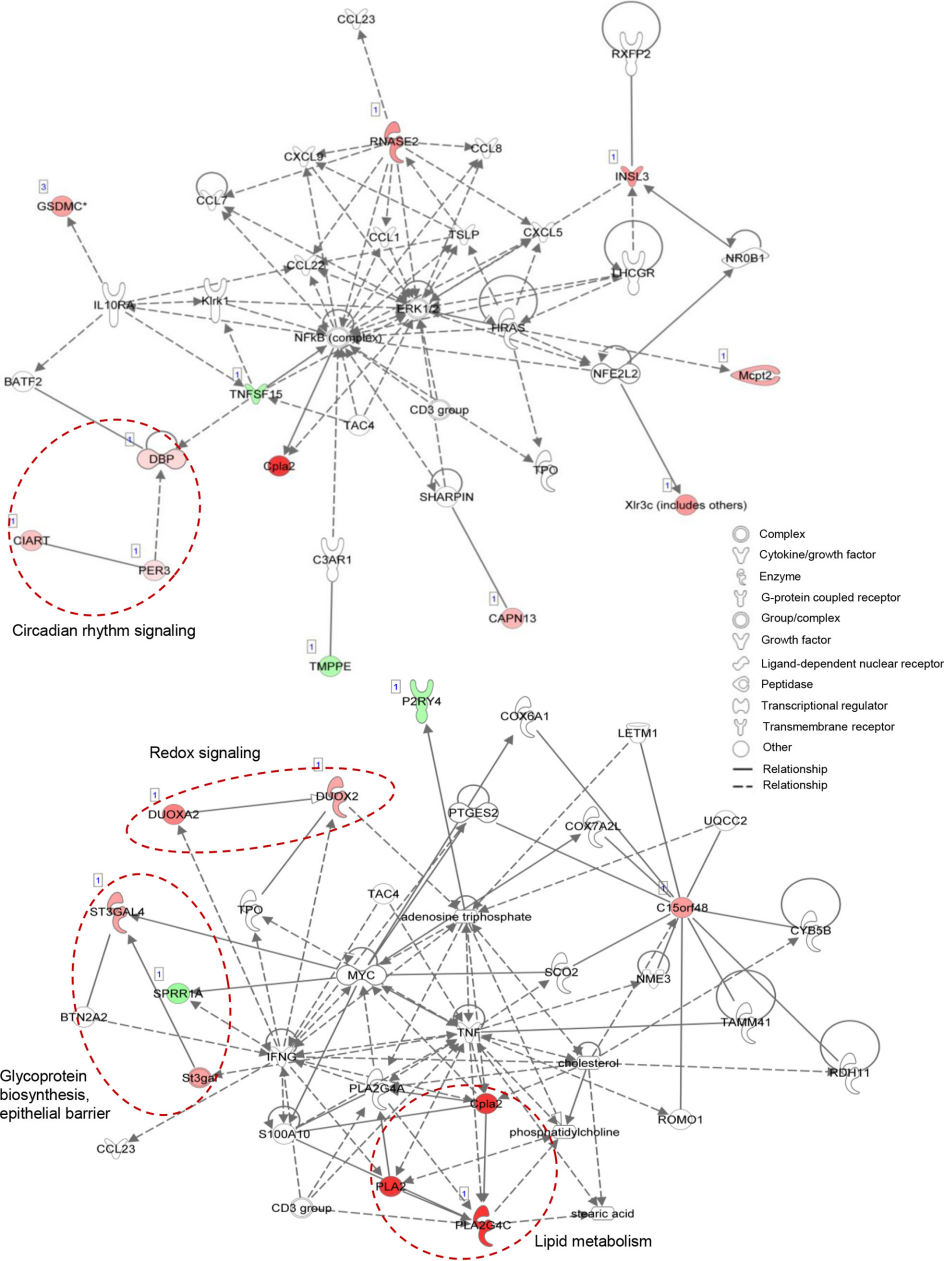
Signaling and molecular network mapping of genes differentially expressed in the ileal transcriptome of NCFM-colonized versus germfree mice (24 genes) using the Ingenuity Pathway Analysis software. Twenty of the genes were mapped to two major networks, with network 1 (top) associated with cellular movement, hematological system development and function, and immune cell trafficking, and network 2 (bottom) involved in lipid metabolism, molecular transport and small molecule biochemistry. Up- and downregulated genes are shown by red and green symbols, respectively, and data set molecules that are not in the query are shown by white symbols. Selected group of genes with common biological functions are indicated by dashed red circles.

To further investigate the mechanistic impacts of NCFM colonization on the host small intestinal epithelium, mRNA-seq expression profiling was performed on the ileal tissues from the same NCFM-treated and germfree control mice groups (see Table S1 at Dryad, https://doi.org/10.5061/dryad.mcvdncjzr). Twenty-four genes were detected as differentially regulated by ≥1.5-fold with statistical significance between the two groups (FDR-adjusted *P* value of ≤0.05), with 17 and 7 genes upregulated and downregulated, respectively, in the NCFM-colonized mice (Table 1). Germfree mice have been known to exhibit impaired gut physiology, including epithelial cell proliferation and differentiation, impaired villus angiogenesis, lower cell turnover and wound healing rates, and immature immune system (reviewed in reference 50). Notably, NCFM colonization resulted in the downregulation of *Tnfsf15*, a proinflammatory cytokine and mediator of apoptosis (Fig. 5). The downregulation of TNFSF15 is predicted to inhibit the downstream NF-κB-directed transactivating cascade of inflammatory signaling. Based on pathway analysis, the repression of *Tnfsf15* was predicted as the result from activation of the upstream regulator IL-10 receptor (IL10RA) (see Fig. S3B at Dryad, https://doi.org/10.5061/dryad.mcvdncjzr), which could consequently suppress proinflammatory cytokine production.

**TABLE 1:** Differentially expressed genes in mice mono-colonized with L. acidophilus NCFM compared to germfree mice^*a*^

Gene	Product	GO biological process(es)	DE log_2_ fold change	FDR-adjusted *P* value
Upregulation				
*Pla2g4c*	Phospholipase A2, group IVC (cytosolic, calcium-dependent)	Glycerophospholipid catabolic process	Δ 7.20	6.59E−06
*Duox2*	Dual oxidase 2	Superoxide anion generation	Δ 3.04	4.3E−03
*Duoxa2*	Dual oxidase maturation factor 2	Regulation of inflammatory response	Δ 4.39	1.38E−05
*Rnase2a*	Ribonuclease, RNase A family, 2A (liver, eosinophil-derived neurotoxin)	RNA phosphodiester bond hydrolysis	Δ 3.88	1.39E−02
*Insl3*	Insulin-like 3	Negative regulation of cell proliferation/ apoptotic process	Δ 3.59	8.80E−03
*Xlr3b*	X-linked lymphocyte-regulated 3B	Spermatid development/meiotic cell cycle	Δ 3.53	4.07E−09
*AA467197*		Response to bacterium	Δ 3.44	4.32E−02
*St3gal4*	ST3 β-galactoside α-2,3-sialyltransferase 4	Glycoprotein biosynthesis process, sialylation	Δ 3.27	1.39E−02
*Gsdmc2*	Gasdermin C2	Programmed cell death, pyroptosis	Δ 3.19	6.56E−03
*Gsdmc3*	Gasdermin C3	Programmed cell death, pyroptosis	Δ 3.26	6.67E−03
*Gsdmc4*	Gasdermin C4	Programmed cell death, pyroptosis	Δ 3.10	6.67E−03
*Mcpt2*	Mast cell protease 2	Proteolysis	Δ 3.05	3.69E−02
*Capn13*	Calpain 13	Proteolysis	Δ 2.44	1.01E−02
*Gm2000*			Δ 2.00	1.24E−03
*Ciart*	Circadian-associated repressor of transcription	Rhythmic process, circadian regulation of gene expression	Δ 2.03	6.56E−03
*Dbp*	D site albumin promoter-binding protein	Rhythmic process	Δ 1.35	4.17E−03
*Per3*	Period circadian clock 3	Rhythmic process	Δ 1.08	3.64E−02

Downregulation				
*Asdurf*			∇ −5.33	2.40E−02
*Gm28539*			∇ −5.21	1.38E−05
*Sprr1a*	Small proline-rich protein 1A	Peptide cross-linking, keratinocyte differentiation	∇ −2.33	8.80E−03
*Lancl3*	LanC lantibiotic synthetase component C-like 3		∇ −2.18	3.35E−02
*Tnfsf15*	Tumor necrosis factor (ligand) superfamily, member 15	Activation of cysteine-type endopeptidase activity involved in apoptotic process	∇ −1.92	3.56E−02
*P2ry4*	Pyrimidinergic receptor P2Y, G protein coupled, 4	Regulation of presynaptic cytosolic calcium ion concn, G-protein-coupled receptor signaling pathway	∇ −1.89	6.56E−03
*Tmppe*	Transmembrane protein with metallophosphoesterase domain		∇ −1.88	4.82E−02

a GO, gene ontology; DE, differentially expressed.

Both *Duox2* and *Duoxa2* encoding the subunits of a dominant NADPH oxidase (NOX) involved in redox signaling in the gut epithelium were upregulated in the NCFM-colonized mice. The DUOX2/DUOXA2 constitutes the predominant hydrogen peroxide-producing system localized at the villous tip of the ileal epithelium and consequently plays key roles in epithelial homeostasis, innate immune defense, modulation of microbial colonization, mucin production, and mucosal healing (51–53). Expression of this redox system is microbially driven and was more active in the ilea of conventional mice compared to germfree mice (54). Despite the lack of Duox2 induction by previous monocolonization of germfree mice with commensals of the distal gut such as Escherichia coli, Bifidobacterium longum, and Bacteriodes thetaiotaomicron (52), our present study demonstrated that mono-association of the small intestinal commensal L. acidophilus NCFM in germfree mice was capable of inducing *Duox2/Duoxa2*. This suggests a regulatory role of NCFM on redox signaling in the ileum.

Colonization by L. acidophilus NCFM also induced host β-galactoside α-2,3-sialyltransferase (ST3GAL4) responsible for lipid and protein glycosylation, specifically *O*-glycosylation of small intestinal mucin Muc2 (55). Glycosylation serves to protect the mucin protein core from microbial degradation while also providing a nutrient source and anchoring sites to the resident microbes. Higher expression of ST3GAL4 was previously observed in the small intestines of conventional mice compared to germfree mice, where the latter also exhibited shorter and less abundant Muc2 sialylated glycans in the small intestine (55). These observations suggested a role for microbiota in the development of a normal mucus layer. The induction of *St3gal4* in the present study reflects modulation of the host mucin glycosylation in response to NCFM colonization. Intriguingly, transcripts associated with circadian rhythm signaling and regulation (DBP, PER3, and CIART) were enriched in the NCFM-colonized mice. Studies have indicated that the intestinal microbiota influences gut circadian rhythms and consequently regulates their hosts’ metabolic homeostasis. As such, manipulation of the microbial composition may be a viable strategy to restore circadian rhythm and metabolic homeostasis (56). The significant enrichment of this biological function in the differential transcriptome suggests a potential novel role of NCFM in gut-brain signaling and the restoration of host circadian-metabolic axis, possibly via the production of beneficial metabolites.

### Genome stability of NCFM during gut transit.

Metagenome sequencing of L. acidophilus NCFM populations recovered from all mono-associated mice at the end of study (Fig. 1A) was performed to detect any genetic aberrations and rearrangements potentially driven by *in vivo* gut colonization. Metagenomic analysis did not detect significant sequence variation in noncoding regions. Only single nucleotide polymorphism (SNP) mutations were detected within four genes across all six *in vivo* passage populations (see Fig. S4 at Dryad, https://doi.org/10.5061/dryad.mcvdncjzr). The prominent SNP resulted in a premature stop codon in *lba0132* in NCFM populations from all mice (with read coverages of >10 and variant frequencies of 14 to 43%), corresponding to an average of 0.25 (±0.12) SNP per genome. The *lba0132* gene, which was significantly upregulated in the cecum and colon, encodes a putative AcrR family transcriptional repressor, which acts as a sensor to monitor both environmental cues and their regulated genes, many of which are related to osmotic stress, metabolic homeostasis, bacteriocin production, multidrug resistance, and pathogenesis (57). We hypothesize that the gradual selective inactivation of LBA0132 across NCFM populations in all gnotobiotic mice may represent an evolutionary trend to allow constitutive expression of the regulon, perhaps to enhance the population stress tolerance and environmental fitness through relieving the repressor regulatory activities.

The generation time of L. acidophilus NCFM during colonization in germfree mice is unknown and is presumably highly unsynchronized when considering the washout rate and host self-inoculation of the microbes through coprophagy. The induction of various metabolic pathways and cell division proteins in NCFM from the ileum through the colon suggests that NCFM is physiologically active *in vivo*. Assuming NCFM cells have a lower growth rate *in vivo* compared to laboratory growth conditions and underwent one to five cell division cycles/day throughout the study, we estimated the mutation rate of NCFM (with an average of 0.25 SNP per genome) to be 0.004 to 0.007 SNP/genome/generation. This is a similar magnitude to the mutation rates observed in E. coli grown *in vitro* or transit through mouse gut (58, 59). The observation reflects the overall genomic stability of NCFM, an ideal property for a probiotic microbe and a platform for engineering delivery of biotherapeutics and vaccines. The relatively stable genetic landscape of NCFM may explain its sole isolation source from mammalian hosts and its specialized adaptation to the intestinal environment.

### Conclusions.

Transcriptome studies of L. acidophilus NCFM using a germfree murine model have provided mechanistic insights into the lifestyle and key genetic determinants involved in its gut survival and adaptation. This is the first report describing the transcriptional response of L. acidophilus *in vivo*, concurrently with host intestinal transcriptional responses when mono-associated with L. acidophilus. Strikingly, some of the gut-induced genes were previously characterized by *in vitro* studies and were predicted to be associated with the survival and beneficial effects of NCFM. The outcomes of the current *in vivo* studies solidify our previous *in silico* and *in vitro* experimental platforms by linking genotypes to phenotypes that are relevant for probiotic functionality.

The constitutive high expression of *slpA in vivo* together with phenotypic analysis of an S-layer mutant highlights the indispensable roles of the S-layer in the maintenance of cell shape and expression of other surface-associated proteins that have pleiotropic impacts, including effects on cell viability. Comparative transcriptome analyses of L. acidophilus NCFM from the ileum through the colon revealed core induced gene sets with prominent functions in carbohydrate anabolic and catabolic pathways, amino acid and peptide foraging, nucleotide biosynthetic pathways, and the orchestrated expression of surface proteins predicted to mediate epithelial interactions and immune functions. Spatial expression profiling indicated differential metabolic status and preferences in carbohydrate and amino acid substrate utilization, which were likely shaped by the nutrient landscape along the gut. Canonical stress response-related chaperones, proteases, and DNA repair were not induced, especially in the ileum where the physiological condition is generally inhibitory to microbial activities. This suggests that NCFM has evolved to reprogram its stress adaptation strategies in order to flourish in the gut microenvironments. The antigenic variation of *slp* genes and transcriptional silence of LTA biosynthesis genes in the ileum led to our hypothesis that these immunostimulatory components are coordinately regulated to educate the immune system without eliciting deleterious immune response in the hosts. Despite relatively minimal shifts in the ileal transcriptomes in response to colonization by NCFM, some of the differential regulated gene expression could be related to positive impacts on the host. These include significant downregulation of proinflammatory cytokines IL-12 and TNFSF15, activation of redox signaling that is crucial to the immune defense and gut epithelial development, modulation of mucin glycosylation, and the potential restoration of host circadian-metabolic axis. These findings provide a framework to inform future studies on investigating the probiotic mechanisms of L. acidophilus under complex *in vivo* ecosystems. Research priorities will also be directed to assigning functions to novel proteins induced *in vivo* that are relevant to host immunomodulation, epithelial barrier integrity, gut-brain signaling, and interaction within the microbial gut community. Finally, the observed relative stability of the NCFM genome postcolonization not only establishes the species as a gut specialist but also advocates for its safety as a probiotic microbe. The genomic integrity of NCFM and the *in vivo* inducible genomic “hot spots” revealed from this study further establish a valuable expression platform for genetic engineering of this species for mucosal delivery of biotherapeutics and vaccines.

## MATERIALS AND METHODS

### Bacterial culture conditions.

L. acidophilus NCFM was propagated in MRS broth (Difco) at 37°C statically under ambient atmospheric conditions. For transcriptome sequencing of *in vitro*-grown cells, NCFM was grown in MRS broth, and culture aliquots were harvested at mid-log (optical density at 600 nm [OD_600_] of ∼0.6) and stationary phases (16-h growth, OD_600_ of ∼3.5 to 4.0) by centrifugation at 3,220 × *g* for 5 to 8 min at room temperature. Cell pellets were flash frozen in an ethanol-dry ice bath and stored at −80°C until sample processing for RNA isolation. Two biological replicate cultures were grown and collected for total RNA isolation.

### Mouse colonization and sampling.

Germfree 129S6/SvEv mice (6 males, 3 females; 24 to 25 weeks old) were used for the experiments carried out at the North Carolina State University (NCSU) Gnotobiotic Core Facility. Animal use protocols were approved by the Institutional Animal Care and Use Committee of NCSU. Mice were maintained in cages in germfree flexible film isolators housed in a room with cycles of 12 h of light and darkness and were provided access to a standard diet (Prolab RMH 3500; LabDiet) and water *ad libitum*. Colonization study design and sampling workflow were conducted as shown in Fig. 1A.

### RNA isolation.

RNA isolation strategies and protocols were developed in order to recover high quality bacterial RNA from intestinal tissue samples, as well as total RNA from intestinal tissues, for downstream RNA-seq and RT-qPCR analyses. Optimized protocol for bacterial RNA isolation from intestinal tissues involved prior physical separation of bacterial cells from the intestinal tissue to minimize the presence of host RNA in the bacterial RNA samples. This step is followed by parallel purification of the bacterial and host total RNA. Briefly, segments of duodenum, jejunum, ileum, colon (each of which is approximately 2 cm in length), and cecum (all tissues were preserved in RNAlater reagent immediately after harvested) were transferred to sterile petri dishes with RNase-free forceps and longitudinally incised to expose the mucosal epithelium. Mucosa-adhered bacteria were recovered along with luminal content by gentle scrapping of the mucosa using sterile, disposable cell scrappers (Nunc, 32 cm; Thermo Fisher Scientific). The luminal and tissue scraping contents were transferred into TRI reagent for bacterial RNA isolation, while the scraped tissue samples were sized to 20 to 50 mg, followed by the addition of 1 ml of TRI reagent for isolation of tissue RNA. Both bacterial and tissue samples were first homogenized by beadbeating with 0.1-mm glass beads and 1.0-mm zirconia beads (Biospec Products), respectively, in a Mini-Beadbeater 16 (Biospec Products) for five or six cycles of 1 min, with 1 min on ice at intervals, to facilitate cell lysis. Total RNA isolation and purification were performed by using the TRI reagent protocol in combination with Qiagen RNeasy Mini or RNeasy Plus Universal minikit with DNase treatment. Two biological replicates of RNA samples were also prepared from L. acidophilus NCFM log-phase and stationary-phase cultures grown in MRS medium (see above). Briefly, aliquots of 10 ml and 4 ml of log- and stationary-phase cells, respectively, were harvested and resuspended in 1 ml of TRI reagent. Cells were disrupted by beadbeating as described above. The homogenates were centrifuged at 14,000 rpm for 10 min at 4°C, and RNA isolation was performed using the Direct-Zol RNA Miniprep kit (Zymo Research) with on-column DNase I treatment. The RNA samples were further treated with TURBO DNase (Thermo Fisher) to remove traces of genomic DNA, followed by a final purification step using the RNA Clean & Concentrator-25 kit (Zymo Research). The absence of genomic DNA in the RNA samples was verified by PCR using gene-specific primers. Total RNA quality was assessed using an Agilent 2100 bioanalyzer (Agilent Technologies).

### mRNA library construction and sequencing.

For transcriptome sequencing of L. acidophilus NCFM recovered from the intestinal segments, bacterial RNA of high quality (based on bioanalyzer analysis) from the ileal tissues was obtained from only two of the six NCFM-fed mice (mouse 1 [M1] and M4; see Table S1 at Dryad, https://doi.org/10.5061/dryad.mcvdncjzr). To maintain a balanced number of biological replicates, two bacterial RNA samples of highest quality isolated from the cecum (M5 and M6) and colon (M5 and M6) were also selected for mRNA-seq expression analysis, along with two biological replicate samples each from the *in vitro* control NCFM (in PBS prior to gavage; day 0/time zero) and MRS-grown log- and stationary-phase cultures. The RNA samples were submitted to the High-Throughput Sequencing and Genotyping Unit of the Roy J. Carver Biotechnology Center at the University of Illinois for mRNA library construction and sequencing. Ribosomal RNAs were first removed with the Ribozero Bacteria kit and the Ribozero Epidemiology kit (Illumina) for *in vitro* and *in vivo* samples, respectively, followed by construction of mRNA libraries using the TruSeq Stranded Total RNA Library Prep kit (Illumina). The libraries were sequenced on a HiSeq 2500 or a HiSeq 4000 ultrahigh-throughput sequencing system (Illumina) using HiSeq SBS v4 kit and HiSeq 4000 v1 kit, respectively, with single read length of 150 to 160 nucleotides (nt). Fastq files were generated and demultiplexed with the bcl2fastq v2.17.1.14 conversion software (Illumina), and adaptor sequence was trimmed from the raw reads. High quality reads were generated as assessed by using FastQC v.0.11.5.

For mRNA sequencing of the mouse ileal tissue RNA, purified RNA samples from each of three germfree mice (M7, M8, and M9; see Table S1 at Dryad, https://doi.org/10.5061/dryad.mcvdncjzr) or NCFM-colonized mice (M1, M4, and M5) were submitted to the above sequencing facility. RNA-seq libraries were prepared with Illumina’s TruSeq Stranded mRNAseq Sample Prep kit (Illumina), quantitated by qPCR, and sequenced on one lane for 151 cycles from each end of the fragments (pair-end read length of 150 nt) on a NovaSeq 6000 System using a NovaSeq SP reagent kit. Fastq files were generated and demultiplexed with bcl2fastq v2.20 conversion software (Illumina), and adaptors were trimmed from the 3′ ends of the reads. A mean of 87.8 million reads per sample was generated (see Table S9 at the above URL), with high quality of reads as assessed by FastQC.

### RNA-seq bioinformatic and statistical analyses.

Mapping of reads to the L. acidophilus NCFM genome (GCF_000011985.1_ASM1198v1) or the GRCm38 Mus musculus gene transcripts and differential gene expression analyses for both bacterial and host samples were performed using the CLC Genomics Workbench v12.0 RNA-seq Analysis tool set (Qiagen). Briefly, library size normalization was performed using the TMM (trimmed mean of M values) method (60) in the RNA-seq Analysis tool, which was then used as part of the per-sample normalization. After TMM factors were calculated for each sample, the TMM-adjusted log CPM counts were calculated. Cross-sample normalization was performed with Gaussian normalization (Z-score normalization) for each gene, where the counts for each gene were mean centered and scaled to unit variance. Expression values of bacterial and host genes were based on normalized transcripts per million (TPM) and reads per kilobase per million (RPKM) mapped reads, respectively. Statistical differential expression test was performed with multifactorial statistics based on the fit of a generalized linear model (GLM) with a negative binomial distribution, similar to EdgeR (61) or DESeq2 (62). Specifically, under the option “Differential Expression for RNA-seq,” the Wald statistical test was used in the “Against control group” comparison (control group = NCFM *in vitro* in PBS day 0/time zero for NCFM *in vivo* transcriptome comparison; control group = germfree for host ileal transcriptome comparison). Fold changes were calculated from the GLM, which corrects for differences in library size between the samples and the effects of confounding factors. Differentially expressed genes with a false discovery rate (FDR)-adjusted *P* value of 0.05 or less were considered statistically significant. Details on CLC Genomics Workbench RNA-seq Analysis statistical methods are documented in the software manual (63). Pathway analysis and prediction of host genes induced in response to NCFM colonization were performed using Ingenuity Pathway Analysis (IPA) software (Qiagen).

### RT-qPCR analysis of host gene expression.

Total RNA was isolated from the intestinal segments (duodenum, jejunum, ileum, cecum, and colon) of germfree mice (*n *=* *3; M7, M8, and M9) and gnotobiotic mice (*n *=* *3; M1, M4, and M5) (total of 30 samples; see Table S1 at Dryad, https://doi.org/10.5061/dryad.mcvdncjzr) as described above and treated with Turbo DNase (Thermo Fisher Scientific) to remove any contaminating DNA (see above). All oligonucleotide primers used for RT-qPCR were listed in Table S8 at the above URL. The absence of genomic DNA in purified RNA samples was verified by PCR using primers specific for the M. musculus F11r gene, which will generate a 314-bp amplicon in the presence of genomic DNA. For a positive control, 200 ng of NIH 3T3 mouse genomic DNA (NEB) was used in the PCRs. The iScript One-step RT-PCR kit with SYBR green (Bio-Rad Laboratories, Hercules, CA) was used for RT-qPCR according to the manufacturer’s suggestions, with each reaction mixture scaled down to a total volume of 25 μl containing 50 ng of RNA template and a final concentration of 300 nM of each primer. For each gene target, two technical replicates were performed for each of the three biological replicates representing germfree or NCFM-colonized samples, for each of the five intestinal segment RNA samples. RT-qPCR was performed with an iCycler MyiQ single-color detection system (Bio-Rad). Data were analyzed using iCycler MyiQ software v1.0 (Bio-Rad). The number of threshold cycles per well was determined using the auto-calculated “threshold cycle calculation” and “PCR baseline subtracted curve fit” analysis mode. To generate standard curves from known concentrations of PCR product, 2 μg of each purified, DNase-treated RNA sample was reverse transcribed using the SuperScript IV First-Strand cDNA synthesis system (Invitrogen) according to the manufacturer’s suggestions in a 20-μl reaction mixture. Aliquots of each resulting cDNA pool (2 μl per 50-μl PCR mixture) were used as the templates for generating PCR products using gene transcript-specific primer pairs (see Table S8 at the above URL). Transcript copy numbers were quantified from standard curves generated from known concentrations of PCR products. The correlation coefficients for the standard curves range from 0.988 to 0.999.

### Metagenome sequencing of NCFM post-murine colonization.

A portion of the fecal sample collected on day 7 (Fig. 1A) from each of the six NCFM-colonized mice was inoculated aseptically into MRS broth medium and grown for 16 h at 37°C under anaerobic conditions (ca. 20 to 30 generations). The inoculum culture of L. acidophilus NCFM used for intragastric gavage was also included as a baseline control to detect any genome change resulting from gut passage. Cell pellets from 10-ml aliquot cultures were submitted to Corebiome Inc. sequencing facility for genomic DNA extraction and metagenome sequencing under the StrainView platform. Briefly, genomic DNA was extracted using MO Bio PowerFecal (Qiagen) automated for high throughput on QiaCube (Qiagen), with beadbeating in 0.1-mm glass bead plates. Purified genomic DNA was quantified with Qiant-iT Picogreen dsDNA assay (Invitrogen). Libraries were prepared by a procedure adapted from the Nextera Library Prep kit (Illumina) and sequenced on an Illumina NextSeq with paired-end reads of 2x150 nt using a NextSeq 500/550 High Output v2 kit (Illumina). Reads were filtered for low quality (Q-score < 20) and length (<50), and adaptor sequences were trimmed using cutadapt v1.15. For each of the seven samples, reads (ranging from 2.5 million to 3.7 million of paired-end reads) were subsequently mapped to NCFM reference genome (GenBank accession number NC_006814) using both Geneious mapper and Bowtie2 mapper (64) with default settings, followed by detection of sequence variations using Geneious Find variations/SNPs tool. SNPs detected on overlapping region of paired reads were counted as single read coverage.

### Construction of L. acidophilus NCFM *slpA* and *slpB* double mutant (NCK1973B).

An isogenic deletion mutant of the silent *slpB* gene was first constructed by using the *upp*-based counterselective gene replacement system (9). An in-frame 1,170-bp deletion within *slpB* (*lba0175*) was constructed by first amplifying DNA segments of 743 bp and 757 bp flanking the regions upstream and downstream of the deletion target using primers slpB1/slpB2 (GAA ATA GGA TCC CAG CTA TCA GCC TTC AT, AGA TAC AGC AGA AGC AA) and slpB3/slpB4 (TTG CTG CTT CTG TAT CTT TGA AGA AGG GTG AAG TTG T, TAA AGT AGA GCT CTG ATA GGA AAG GTG CTC AAT), respectively. Purified PCR products of both fragments were fused and amplified to generate copies of deletion alleles via overlap extension PCR with 10 ng of each PCR product as the amplification template in a 50-μl PCR mixture using primer pair slpB1/slpB4 with 25 amplification cycles. Construction of a recombinant integration plasmid, designated pTRK957, and the recovery of double recombinants were performed as previously described (9). Deletion mutants were screened by colony PCR using primer pair slpB5/slpB6 (TTC GTT GCA TCA GCA TAA G, GTG TAG TAT TGC CGA TAA CAG). In-frame deletion of *slpB* in one of the recovered mutants, designated NCK1964, was confirmed by DNA sequencing. Several attempts to construct a *slpA* deletion mutant consistently yielded Δ*slpA* recombinants that also carried the wild-type *slpA* allele, indicating the presence of a mixed population due to selective pressure of the *slpA* gene. Therefore, the *slpA* gene was inactivated by an insertion mutation using pTRK826 (15) in the NCK1964 mutant background using plasmid integration procedures described previously (8). Insertional inactivation of the *slpA* gene was confirmed by PCR and DNA sequencing at the plasmid integration site. The resulting *slpAB* double mutant, designated NCK1973B, was propagated in MRS medium containing 2 μg/ml of erythromycin to maintain the plasmid integration within *slpA* in subsequent functional studies. Procedures for stress challenge assays, survival in simulated gastric juice, adhesion to solvents, mucin, extracellular matrix components fibronectin, laminin, and collagen, and dendritic cell coincubation assays for cytokine analysis were performed as previously described (9, 23, 31). For scanning electron microscopy (SEM), mid-log-phase cells grown in MRS broth were submitted to the Center for Electron Microscopy (CEM) at North Carolina State University for sample processing. Samples were viewed with a JEOL JEM-5900LV SEM at 15 kV. Images were acquired digitally using a JEOL Digital Scan Generator at a resolution of 1,280 × 960 pixels.

### Data availability.

RNA-seq data of L. acidophilus NCFM (*in vivo* and *in vitro* conditions) have been deposited in the NCBI Short Read Archive (SRA) database under the BioProject identifier (ID) PRJNA450639 (accession numbers SRR7016095 to SRR7016104) and PRJNA450642 (SRR7016063 and SRR7016064). Mouse ileal tissue mRNA-seq data have been deposited under the BioProject ID PRJNA566446 (SRR10149701 to SRR10149706). Metagenome sequencing data of NCFM after murine gut passage have been deposited in the SRA database under BioProject ID PRJNA573058 (SRR10153075 to SRR10153081). Supplemental material can be found at Dryad, https://doi.org/10.5061/dryad.mcvdncjzr.
